# SGLT2 Inhibitors and Curcumin Co-loaded Liposomal Formulations as Synergistic Delivery Systems for Heart Failure Therapy

**DOI:** 10.3390/pharmaceutics17080969

**Published:** 2025-07-26

**Authors:** Bianca-Ștefania Profire, Florentina Geanina Lupașcu, Alexandru Sava, Ioana-Andreea Turin-Moleavin, Dana Bejan, Cristian Stătescu, Victorița Șorodoc, Radu-Andy Sascău, Laurențiu Șorodoc, Mariana Pinteala, Lenuța Profire

**Affiliations:** 1 Faculty of Medicine, “Grigore T. Popa” University of Medicine and Pharmacy of Iasi, 16 University Street, 700115 Iași, Romania; bianca-stefania.profire@umfiasi.ro (B.-Ș.P.); cristian.statescu@umfiasi.ro (C.S.); victorita.sorodoc@umfiasi.ro (V.Ș.); radu.sascau@umfiasi.ro (R.-A.S.); laurentiu.sorodoc@umfiasi.ro (L.Ș.); 2 Faculty of Pharmacy, “Grigore T. Popa” University of Medicine and Pharmacy of Iași, 16 University Street, 700115 Iași, Romania; alexandru.i.sava@umfiasi.ro (A.S.); lenuta.profire@umfiasi.ro (L.P.); 3 “Petru Poni” Institute of Macromolecular Chemistry, 41A Grigore Ghica-Voda Alley, 700487 Iasi, Romania; moleavin.ioana@icmpp.ro (I.-A.T.-M.); bejan.dana@icmpp.ro (D.B.); pinteala@icmpp.ro (M.P.); 4 Institute for Cardiovascular Diseases “Prof. Dr. George I.M. Georgescu”, 50 Carol I Boulevard, 700503 Iași, Romania; 5 “Sf. Spiridon” Clinical Emergency Hospital, 1 Independence Boulevard, 700111 Iași, Romania

**Keywords:** SGLT2 inhibitors, curcumin, liposomes, heart failure

## Abstract

**Background/Objectives**: As novel synergistic strategy for heart failure (HF), this study explores the formulation and characterization of liposomal systems co-loaded with SGLT2 inhibitors (dapagliflozin—DAPA and empagliflozin—EMPA) and curcumin (Cur). **Methods**: To enhance liposomal membrane stability and achieve sustained, controlled drug release, oleanolic acid (OA) was incorporated into the lipid bilayer, while the liposomal surface was coated with polyvinylpyrrolidone (PVP). **Results**: The resulting liposomes exhibited favorable physico-chemical properties (particle size ~170 nm, low PDI, negative zeta potential), high encapsulation efficiencies (up to 97%), and spherical morphology as confirmed by STEM. XRD and DSC analyses indicated successful API incorporation and amorphization within the lipid matrix, while PVP coating provided slight improvements in thermal stability. Trehalose proved to be an effective cryoprotectant, preserving liposome integrity after freeze-drying. In vitro release studies demonstrated sustained and delayed drug release, especially in PVP-coated and OA-containing formulations. **Conclusions**: All these findings highlight the promise of PVP-coated, OA-stabilized liposomal formulations co-loaded with SGLT2 inhibitors and Cur as biocompatible, multifunctional platforms for targeted HF therapy.

## 1. Introduction

Heart failure (HF) is a debilitating clinical syndrome with significant and continuously increasing morbidity and mortality, affecting more than 60 million individuals worldwide [1]. A classification of HF disease includes HF with reduced ejection fraction (HFrEF; EF ≤ 40%), HF with mildly reduced ejection fraction (HFmrEF; EF 41–49%), and HF with preserved ejection fraction (HFpEF; EF ≥ 50%). A more common form of HF is HFpEF [2].

Despite significant therapeutic advances made to date, HFpEF currently lacks an effective, evidence-based therapy that demonstrably extends the life span of HF patients. Dapagliflozin (DAPA) and empagliflozin (EMPA) are potent, competitive, and reversible human SGLT2 inhibitors, used mainly for the therapy of type 2 diabetes (T2D) [3]. By blocking the sodium-glucose co-transporters (SGLTs), primarily located on the proximal renal tubule, SGLT2 inhibitors decrease the renal reabsorption of glucose simultaneously with an increase in its urinary excretion. Consequently, the level of blood glucose decreases [4]. In 2022, SGLT2 inhibitors were added to HF guidelines, based on the strong evidence showing that they significantly reduce cardiovascular mortality and the risk of hospitalization for HF [5]. In particular, it was demonstrated that EMPA reduced the risk of cardiovascular mortality or hospitalization for HF in adult patients with HFpEF regardless of the presence or absence of diabetes [6]. The main problem of SGLT2 inhibitors, mainly EMPA and DAPA, is their low water solubility (0.173 mg/mL for DAPA, 0.28 mg/mL for EMPA), which directly affects the permeability/dissolution features and, implicitly, their bioavailability [7].

Nowadays, nanotechnology is an attractive technology to improve the solubility, efficacy, and bioavailability of poorly soluble drugs [8]. Liposomes (LPs), as lipid-based nanocarriers with a high capacity to encapsulate hydrophilic, hydrophobic, and amphiphilic drugs, have found wide-ranging applications in various fields, including pharmaceuticals, biomedicine, food, and nutraceuticals [9,10]. Phosphatidylcholine (PC) and cholesterol (Col) are the principal components of liposomal matrix, playing a pivotal role in the stability of LPs and in the release profile of the encapsulated drugs. PC is known to enhance the membrane fluidity, but at the same time, the saturated/unsaturated fatty acid acyl chains from its structure introduces steric hindrance, thereby impeding close molecular packing. This results in reduced van der Waals forces and diminished hydrophobic interactions, ultimately affecting membrane cohesion [11,12]. Moreover, it is reported that hydrophobic drugs require a more rigid liposomal membrane to prevent faster drug release and also offer protection against degradation in the gastro-intestinal (GI) tract [11].

In recent years, several triterpenes and steroids have been investigated for their ability to form LPs in combination with PC. This approach aims to enhance the stability of the liposomal formulations, prolong the release time of the drugs, and improve the GI stability of LPs [13]. For example, when Col is added to PC, a decrease in membrane fluidity was noted. This is due to the insertion of the rigid hydrophobic fragment between the lipid chains of PC, which induces a packing effect [11].

Other researchers reported that pentacyclic triterpenes (TTPs) modulate the properties of the lipid membrane. By adding TTPs to saturated phospholipids, such as dipalmitoyl phosphatidylcholine, LPs with increased membrane stability were obtained [13,14].

Oleanolic acid (OA) is a natural TTP that can be deeply incorporated into unsaturated lipid bilayers in order to promote lipid packing, decreasing the membrane fluidity and increasing the stability of liposomal membrane [15].

This study reports the formulation and characterization of new liposomal formulations containing SGLT2 inhibitors (DAPA and EMPA) and curcumin (Cur) as a synergistic therapeutic strategy for HF. The innovation of this approach stems from the co-encapsulation of active pharmaceutical ingredients (APIs) within a liposomal matrix, enabling SGLT2 inhibitors and Cur to mitigate cardiomyocyte apoptosis through distinct yet complementary mechanisms. According to the current HF guidelines, SGLT2 inhibitors confer cardioprotective effects, while Cur exhibits potent antioxidant and anti-inflammatory properties [16]. The detrimental impact of reactive oxygen species (ROS) on the cardiovascular system is well established: ROS induce electrophysiological disturbances, impair myocardial contractility by altering proteins involved in excitation–contraction coupling, and promote myocardial fibrosis by increasing tissue inhibitors of metalloproteinases and reducing the expression of matrix metalloproteinases [17]. Moreover, the use of LPs as delivery systems enhances the pharmacokinetic profile of the encapsulated APIs, improving their stability, bioavailability, and therapeutic potential.

To enhance liposomal membrane stability and achieve sustained, controlled drug release, OA was incorporated into the lipid matrix, while the liposomal surface was coated with polyvinylpyrrolidone (PVP), as a protective agent. The formulated LPs were characterized in terms of physical parameters (particle size, polydispersity index, zeta potential), encapsulation efficiency, stability, thermal behavior, and drug release profile.

## 2. Materials and Methods

### 2.1. Materials

L-α-phosphatidylcholine (95%) (PC Egg, Chicken) and cholesterol (powder ≥99%) were supplied by Avanti Polar Lipids Inc. (Alabaster, AL, USA). D-(+)-Trehalose dihydrate from Saccharomyces cerevisiae, sucrose for microbiology (ACS reagent ≥99.0%), chloroform (CHCl_3_), ethanol (absolute, 99.5%), Polysorbate 80, polyvinylpyrrolidone (MW 40,000), curcumin (98%), and pepsin from porcine gastric mucosa (cryst. lyophilized 10 FIP-U/mg for biochemistry) were obtained from Sigma Aldrich (St. Louis, MO, USA). Empagliflozin (EMPA, ≥97%) and dapagliflozin (DAPA, ≥97%) were obtained from BLD Pharmatech GmbH (Reinbek Germany). Oleanolic acid (OA, ≥95%) was obtained from Cayman Chemical (Ann Arbor, MI, USA).

### 2.2. Preparation of APIs-LPs/PVP-APIs-LPs

#### 2.2.1. Preparation of Blank LPs

The blank LPs were prepared in order to establish the optimal composition and physico-chemical features of LPs that will be used for APIs encapsulation. These parameters influence both the effectiveness of embedding of APIs and the ability of LPs to cross the biological membranes. LPs were prepared by thin-film hydration technique followed by extrusion/non-extrusion and purification through PD-10 desalting columns packed with Sephadex G-25 resin (Cytiva, Global Life Science Solutions Operations, Buckinghamshire, UK) [15,18]. The PC (30/20 mg) and Col (5 mg) were dissolved in 1 mL CHCl_3_:C_2_H_5_OH (2:1, *v*/*v*) by vigorously stirring in a 10 mL round bottom flask

**Figure 1:**
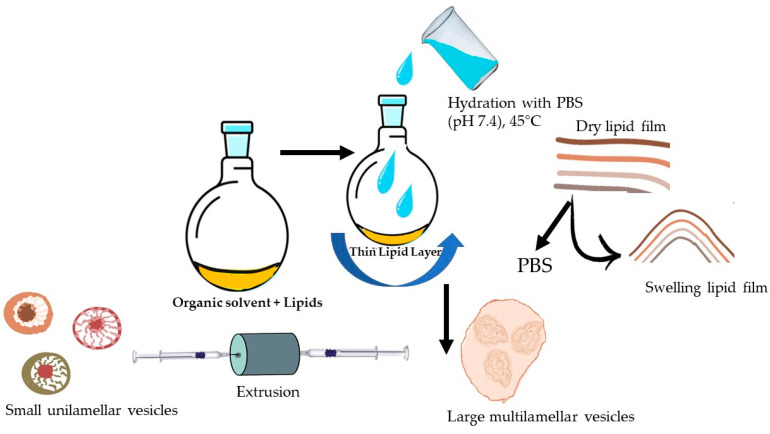
Preparation of LPs through thin-film hydration technique.

To form the lipid film, the solvents were completely removed using a rotary vacuum evaporator (LabTech, EV 400-V, USA) under reduced pressure for 1 h. The flask was kept overnight in a vacuum oven (Memmert GmbH, UF 55, Laboratorium, Germany) for the complete removal of residual solvent. Subsequently, the lipid film was hydrated with PBS (400 µL, pH 7.4) under gentle heating and stirring. This process resulted in swelling and the detachment of lipid sheets during agitation, leading to the self-assembly of large multilamellar vesicles (LMVs). Before extrusion, the LMV suspension was subject to freeze–thaw cycles in liquid nitrogen/+45 °C (9–10 cycles) to improve the homogeneity of the size distribution of LPs and to increase the effectiveness of the extrusion process. Through extrusion, which is based on the input of mechanical energy, the particle size of LPs is reduced. LP suspension was passed through two polycarbonate membranes with a pore size of 400 and 200 nm, 12 times above Tc, using an Avanti Mini Extruder (Avanti Polar Lipids, INC., Alabaster, AL, USA). The LP preparation method is briefly presented in Figure 1. The LPs that presented the most favorable features, in terms of particle size (PS) and stability, were chosen for API encapsulation.

#### 2.2.2. Preparation of APIs-LPs

To prepare the APIs-LPs, the method, presented in Section 2.2.1., was applied. PC and Col were dissolved in 1 mL CHCl_3_:C_2_H_5_OH (2:1, *v*/*v*) in a 10 mL round bottom flask, and then the APIs, dissolved in the proper solvent (ethanol for DAPA, EMPA, Cur; CHCl_3_-ethanol 3:1 for OA), was added.

To establish the optimal formulation, both co-loaded APIs-LPs (EMPA/DAPA–Cur/OA-LPs) and single-loaded API-LPs (EMPA/DAPA/Cur/OA-LPs) were prepared using various API:PC ratios alongside blank LPs. The compositions of the single- and co-loaded LPs, including the concentrations of Col, PC, APIs, and their respective API:PC ratios, are detailed in Table 1.

#### 2.2.3. Preparation of PVP-APIs-LPs

To improve the stability of the APIs-LPs, their surface was covered with PVP. Briefly, PVP was dissolved in distilled water (1%) and stirred for 2 h. Then, 400 µL was used to hydrate the thin lipid film, and the PVP-APIs-LPs were separated using size exclusion chromatography. Finally, PVP-APIs-LPs were transferred onto a Sephadex column that was pre-equilibrated with PBS (pH 7.4) and eluted with PBS [19]. The collected PVP-APIs-LPs dispersions were stored at 4 °C for further analysis.

### 2.3. Characterization of LPs

#### 2.3.1. The Physical Parameters

After preparation, the LPs (blank LPs, APIs-LPs, PVP-APIs-LPs) were analyzed in terms of particle size (PS), zeta potential (ZP), and polydispersity index (PDI), by Dynamic Light Scattering (DLS), using an Easier Nano ZS90 instrument (Malvern Instruments, Malvern, UK). The LPs were diluted with ultrapure water in a ratio of 1:30 (*v*/*v*), and the measurements were performed at a temperature of 24 °C with a scattering angle of 90°.

#### 2.3.2. Scanning Transmission Electron Microscopy (STEM)

The images were obtained using a STEM 3+ detector (Bright-Field Mode) at an accelerating voltage of 30 kV by analyzing a drop of LPs solution that was deposited on 300-mesh-size, carbon-coated copper grids and dried at room temperature.

#### 2.3.3. X-Ray Diffraction (XRD)

Freeze-dried APIs-LPs and APIs powder were analyzed using a Rigaku SmartLab X-ray diffractometer (Rigaku Corporation, Tokyo, Japan) in Bragg–Brentano geometry with a Cu anode (with an X-ray wavelength of 1.5406 Å) within an angular range of 2–60°, with a scanning step of 0.02° and a recording rate of 3°/min.

#### 2.3.4. The Encapsulation Efficiency (EE)

##### 2.3.4.1. HPLC Method

The standard APIs (EMPA, DAPA, Cur) content was determined using HPLC Shimadzu Nexera LC-40-XR system (Schimadzu H.mbH Korneuburg, Kyoto, Japan) equipped with an autosampler (SIL 40 XR), SPD-40V series UV-Vis, and RF-20Axs fluorescence detector. Chromatographic separation of the APIs was performed in a C18 column (4.6 × 150 mm, Atlantis dC, 5 μm) using three mobile phases: A (water/formic acid—99.9/0.1, *v*/*v*), B (acetonitrile), and C (methanol). Before use, the solvents were filtered through a 0.22 μm filter and degassed by ultrasonication. The injection sample was 10 μL, the run time was 9 min in isocratic mode (1 mL/min), and the optimal mobile phase ratio was A:B:C = 45:42:13 (*v*/*v*). The column temperature was kept at 30 °C during the chromatographic separation with fluorescence detection for Cur (λ_ex_ = 430 nm and λ_em_ = 550 nm) and for DAPA/EMPA (λ_ex_ = 278 nm and λ_em_ = 303 nm). The qualitative and quantitative analysis of the APIs (EMPA, DAPA, Cur) was carried out based on the retention times and peak areas R. For the peak integration, the LabSolutionDB software, version 6.106SP1 was used.

In order to quantify the concentration of APIs, the standard curve for each APIs was plotted. For this, stock standard solutions of APIs (EMPA, DAPA, Cur) were prepared by dissolving an appropriate amount of each in methanol to give a final concentration of 2000 ppm. From each stock solution, a serial dilution in methanol was prepared to obtain different concentrations of 0.5, 1.5, 2.5, 5, 10, 20, 40, 60, 80, and 100 ppm. From each serial dilution, an aliquot of 10 µL was injected.

A calibration curve is a plot of the area under the peak (AU) to the external standard as a function of the drug concentration [20]:

AU = Slope × Concentration + Intercept

The slope and the intercept were calculated based on AU and the concentration of APIs.

##### 2.3.4.2. Encapsulation Efficiency (EE) of APIs into Liposomal Matrix

The EE (%) of the APIs into the LPs was quantified using the HPLC method described in Section 2.3.4.1. Briefly, 0.5 mL of purified LPs were lyophilized and diluted with 1 mL of methanol and sonicated for 10 min in an ultrasonic bath (Elmasonic P, Germany) at room temperature. Afterwards, the LP suspension was centrifuged at 15,000 rpm for 5 min and the supernatant was passed through a 0.22 µm filter. The API content of the filtrate was quantified using the following formula [21]:

EE% = W_1_/W_0_ × 100

where W_0_ is the amount of APIs used for loading in LPs; W_1_ is the amount of APIs loaded in LPs.

### 2.4. Stability of LPs

#### 2.4.1. Storage

The storage stability parameter was determined by storing APIs-LPs in an aqueous medium at 4 °C and 24 °C for 3 weeks. A volume of 5 mL of freshly prepared APIs-LPs were transferred into 10 mL brown glass bottles. PS, PDI, and ZP were measured at specific time intervals after preparation (0, 7, 14, 28 days).

#### 2.4.2. Osmotic Stress

APIs-LPs were kept under 0.6% NaCl solution (hypotonic) and 1.2% NaCl solution (hypertonic) for 10 min at room temperature and the changes in PS, PDI, and ZP were noted, using DLS [22]. For comparison, the behavior in PBS 7.4 was also performed, using similar experimental conditions.

#### 2.4.3. Freeze-Drying Stress

APIs-LPs and their PVP-coated counterparts were immersed in sucrose and trehalose solutions as cryoprotectants, using the sugar-to-lipid ratios of 2:1 (*w*/*w*) and 1:1 (*w*/*w*), respectively. The mixtures were stirred at 150 rpm for 1 h. Then, the samples were frozen at −20 °C overnight and lyophilized (LabTech^®^ rotary evaporator, EV 400-V, China) at −20 °C under pressure for 10 h. The resulting powders were dispersed in deionized water (2 mL), heated to 45 °C and vortexed in three cycles (30 s) (Vortex V-1 plus, Biosan SIA, Riga, Latvia), followed by a treatment in an ultrasonic water bath for 3 cycles of 60 s each (Elma, Elmasonic P 30H, Elma Schmidbauer GmbH, Germany). LPs without cryoprotectants were used as controls. The effects of the cryoprotectants (sucrose and trehalose) on the physical characteristics in terms of PS, PDI, and ZP were noted, using DLS [18,23].

### 2.5. Thermal Analysis

#### 2.5.1. Thermogravimetry (TG)/Differential Thermal Analysis (DTA)

The thermal behavior of the LPs was investigated using STA 449 F1 JUPITER equipment (Netzsch, Germany). The lyophilized LPs samples were placed into alumina crucibles and subjected to heating within a defined range of 30 °C to 700 °C, with a heating rate of 10 °C/min, under an inert dry nitrogen atmosphere. Prior to each measurement, an isothermal segment at 30 °C was placed for 5 min in order to restore the atmospheric equilibrium in the furnace chamber. The collected data were processed using a NETZSCH PROTEUS software, version 5.0 4.2 (Netzsch, GmbH, Selb, Germany).

#### 2.5.2. Differential Scanning Calorimetry (DSC)

The phase transitions of the lyophilized LPs samples were investigated using a differential scanning calorimeter (DSC 200 F3 MAIA, Netzsch, Germany) from −50 °C to 300 °C (depending on the thermal stability of the sample), under nitrogen protection, at a heating rate of 10 K/min, with purge gas flow rates of 50 mL/min and protective gas flow rates of 100 mL/min. Samples were placed into alumina DSC pans and weighted at an ENTRIS224l-1S balance from Sartorius AG (Goettingen, Germany) with a precision of 0.1 mg. At each cycle, for complete freezing of the sample at −50 °C, an isothermal program was applied to the dynamic segment for 2 min in order to restore the atmospheric equilibrium in the DSC chamber. The data obtained on NETZSCH equipment are processed with the NETZSCH PROTEUS software, version 5.0 (Netzsch, GmbH, Selb, Germany).

### 2.6. In Vitro Release Study

For the quantification of the APIs (EMPA, DAPA, Cur) released from PVP-APIs-LPs (PVP-EMPA-Cur-LPs, PVP-EMPA-OA-LPs, PVP-DAPA-Cur-LPs, PVP-DAPA-OA-LPs), the HPLC method presented in Section 2.3.4.1. was applied.

The in vitro release behavior of APIs from LPs was assessed in two simulated fluids: simulated gastric fluid (SGF) and simulated intestinal fluid (SIF), accordingly to the method recommended by United States Pharmacopeia (Apparatus II) with slight modifications [24,25,26]. For comparison, the behavior of APIs (EMPA, EMPA-Cur, DAPA, DAPA-Cur) in SGF and SIF were also studied.

Briefly, a 3 mL sample of PVP-EMPA-Cur-LPs (containing 2.08 mg of EMPA and 5.67 mg of Cur), PVP-EMPA-OA-LPs (containing 2.37 mg of EMPA), PVP-DAPA-Cur-LPs (containing 0.36 mg of DAPA and 6 mg of Cur), PVP-DAPA-OA-LPs (containing 0.36 mg of DAPA), and APIs (equivalent amount in ultrapure water) was placed into a dialysis membrane (Spectra/Por 4, molecular cut-off 12–14 kD, California, CA, US, Canada), which was soaked for 12 h before use. After that, the dialysis membrane was introduced in the stainless-steel basket of the USP standard dissolution apparatus and immersed in a 250 mL hemispherical glass dissolution vessel containing 90 mL SGF or SIF as a release medium. SIF consisted of sodium phosphate monobasic (4.3 g/L), NaOH (0.52 g/L), NaCl (7.75 g/L), taurocholate (3 mM), Tween 80 (3 g/L), 10% ethanol, and NaOH to pH of 7.4. SGF consisted of NaCl (2 g/L), HCl (84 µM), pepsin (3.2 g/L), 25% ethanol, and HCl to pH of 1.5. The spindle was rotated at a constant speed of 100 rpm and the temperature was maintained at 37 ± 0.5 °C. At specified time points, 20 mL of release medium was collected and replaced with an equivalent volume of fresh medium pre-equilibrated at 37 °C to keep a constant release volume. The samples thus collected were centrifuged for 10 min at 15,000 rpm and the supernatant was filtered through a 0.22 μm filter and then 10 μL of sample was injected using HPLC method conditions. Cumulative release (%) was calculated according to the following formula [27]:

$$Cumulative\;release(\%)=\frac{cumulative\;amount\;of\;APIs\;released}{total\;amount\;of\;APIs\;loaded\;into\;LPs}\times 100$$

### 2.7. Data Analysis

All experiments were performed in triplicate and data was presented as mean value ± SD. An unpaired Student’s t-test was performed to compare the two groups and to determine significance and a *p*-value less than 0.05 was considered significant.

## 3. Results and Discussion

### 3.1. Characterization of APIs-LPs

#### 3.1.1. The Physical Parameters

To optimize the LP formulation, several key factors were considered, including the ratio of lipid components (PC:Col), the ratio of APIs to lipid components, and the application of the extrusion process. The physical parameters of the formulated LPs, in terms of particle size (PS), zeta potential (ZP), and polydispersity index (PDI), are presented in Table 2. These parameters are critical in influencing the release profiles of both loaded and co-loaded APIs.

Analysis of the results indicated that these parameters are closely related to lipid composition, API-to-lipid ratio, and the extrusion process. Specifically, for un-extruded LPs, the PS and PDI values were significantly higher than those of the extruded formulation. For example, in the case of LPs@1a, a PS of 1796.00 ± 0.22 nm and a PDI of 0.916 ± 0.017 were recorded, while for the extruded formulation (LPs@1b), the values recorded were 199.60 ± 2.57 nm for PS and 0.351 ± 0.019 for PDI. Additionally, the PS increases slightly with increasing PC:Col ratio, from 191.60 ± 1.85 nm (4:1, LPs@2b) to 199.60 ± 2.57 nm (6:1, LPs@2b, LPs@1b). By incorporation a single API (EMPA, DAPA, Cur, or OA), the PS of the corresponding LP formulations does not vary significantly. For a PC:Col ratio of 6:1, the PS value ranged from 179.40 ± 5.30 nm (Cur-LPs) to 236.80 ± 3.20 nm (OA-LPs). In the case of a PC:Col ratio of 4:1, the values ranged from 166.30 ± 4.70 nm (Cur-LPs) to 221.20 ± 4.30 nm (OA-LPs).

Regarding the ZP, negative values ranging from −10.54 to −31.79 were recorded for both LPs and single APIs-LPs, which ensures some stability, based on repulsive electrostatic forces that prevent particle aggregation [28]. Higher ZP values were recorded for large-sized non-extruded LPs (−31.79 ± 0.57 mV) compared to smaller-sized extruded LPs (−14.86 ± 0.34 mV), explained by the surface composition of LPs. The ZP values of single APIs-LPs were similar to those of the smaller-sized extruded LPs, ranging from −10.54 ± 1.05 mV (DAPA-LPs) to −20.31 ± 3.12 (OA-LPs), indicating that APIs themselves do not significantly affect the surface electrical charge of the LPs.

The results of the physical parameters for the co-loaded APIs-LPs (EMPA/DAPA-Cur/OA-LPs) and their corresponding PVP-coated formulations (PVP-APIs-LPs), prepared using a PC:Col ratio of 4:1 and extruded through 200 nm polycarbonate membranes, are presented in Table 3.

For the co-encapsulated EMPA/DAPA-Cur/OA-APIs-LPs, the PS ranged from 164.2 ± 1.21 nm for EMPA-Cur-LPs(a) to 235.1 ± 5.31 nm DAPA-Cur-LPs(d). The PDI value varied from 0.031 ± 0.01 for EMPA-Cur-LPs(a) to 0.191 ± 0.01 for EMPA-OA-LPs(b), indicating a narrow size distribution.

Additionally, these LP formulations exhibited negative ZP recorded values, ranging from −6.95 ± 0.29 mV for DAPA-Cur-LPs(b) to −10.54 ± 2.05 mV for DAPA-OA-LPs(b). It has been noted that negatively charged LPs remain in the bloodstream longer than the positively charged ones and they are removed slowly from the circulation. This increases the retention time of the drugs, resulting in a longer duration of action [29].

In the case of PVP-coated LPs, slight changes in PS, PDI, and ZP were noted. For example, the PS of PVP-EMPA-Cur-LPs(b) was significantly higher statistically, with a mean value of 289.80 ± 2.01 nm compared to 206.00 ± 12.30 nm for EMPA-Cur-LPs(b) (*p* < 0.01). Regarding the ZP, the values ranged from −5.60 ± 1.34 mV (PVP-DAPA-Cur-LPs(b)) to −7.61 ± 1.52 mV (PVP-DAPA-OA-LPs), a slight change in ZP towards lower negative values compared to uncovered LPs, and this effect is in accordance with the literature, in which the non-ionic polymers cover phosphocholine headgroups in the lipid vesicles through electrostatic interactions, which changes ZP towards almost neutral values [30,31].

#### 3.1.2. Scanning Transmission Electron Microscopy (STEM)

**Figure 2:**
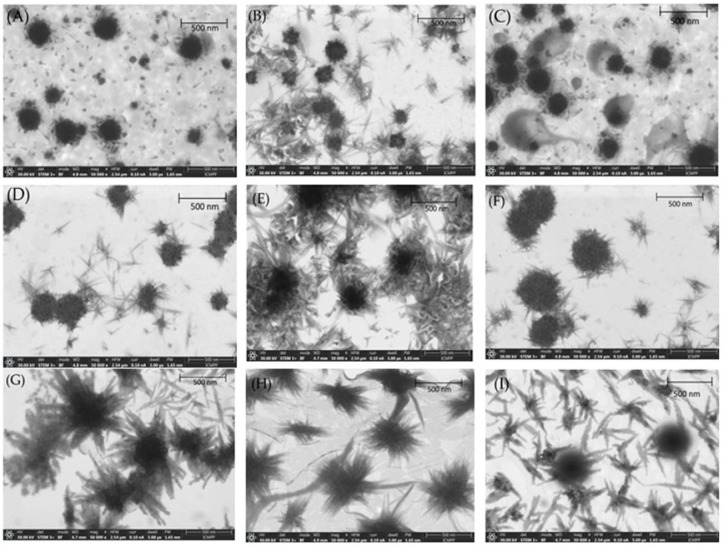
STEM images of co-loaded APIs-LPs: (**A**) EMPA-Cur-LPs; (**B**) EMPA-OA-LPs; (**C**) DAPA-Cur-LPs; (**D**) DAPA-OA-LPs. STEM images of co-loaded PVP-APIs-LPs: (**E**) PVA-EMPA-Cur-LPs; (**F**) PVP-EMPA-OA-LPs; (**G**) PVP-DAPA-Cur-LPs; (**H**) PVP-DAPA-OA-LPs; (**I**) LPs.

The particle size (PS) of API-loaded liposomes (APIs-LPs) and PVP-coated APIs-LPs (PVP-APIs-LPs), along with their stability against aggregation over time and their morphology, were analyzed using scanning transmission electron microscopy (STEM). Both uncoated and PVP-coated APIs-LPs exhibited a nearly spherical shape and were well dispersed, as shown in Figure 2.

STEM images confirmed the good stability of the LPs, attributed to their negative surface charge, which prevents aggregation or precipitation of lipid particles. The PS of LPs via STEM were slightly smaller than those obtained through DLS measurements, due to slight contraction of the LPs during the drying process, which leads to a reduction in overall sizes. DLS analyses measure the hydrodynamic diameters of the particle populations, which include both the particle core and surrounding solvation layer, whereas the STEM measures the physical dimensions of each individual particle in a dry state [32]. Furthermore, a slight increase in PS was noted for PVP-APIs-LPs, confirming the successful coating of APIs-LPs with PVA polymer film. In the case of PVP-APIs-LPs, a rough surface of the particles was also noted, which can be attributed to heterogeneous electrostatic adsorption of the PVP onto the lipid surface.

#### 3.1.3. X-Ray Diffraction (XRD)

**Figure 3:**
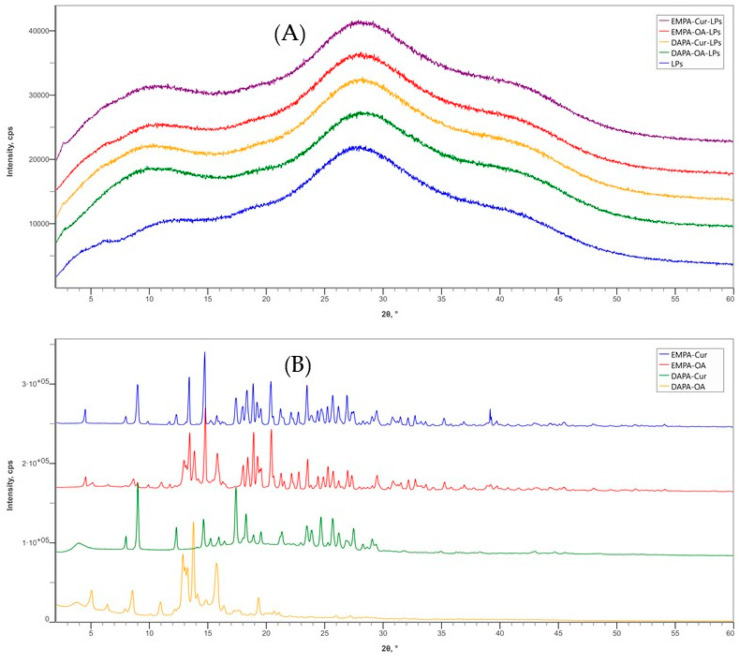
X-ray diffraction patterns: (**A**) LP formulations and (**B**) API physical mixtures.

The XRD spectra of APIs-LPs (EMPA/DAPA-Cur/OA) are presented alongside those of LPs (PC-Col) and the physical mixtures of APIs (EMPA/DAPA-Cur/OA) in Figure 3. This analysis provides important data into the physical state of the APIs incorporated into the LPs. In the diffraction pattern of blank LPs, two broad peaks in the ranges of 7.5° to 13.5° and 25° to 30°, are observed, which are characteristic of phospholipids in lipid bilayer structure.

Our findings are in agreement with the results reported by Cheng et al. [21]. This pattern is also characteristic of APIs-LPs, where no distinct peaks for APIs are observed, indicating that their crystalline form has been transformed into an amorphous one. This change may result from interactions between the APIs and liposomal matrix. Similar phenomena have also been reported for other APIs-LPs [18,33].

For comparison, the XRD spectra of the physical mixture of APIs exhibit strong and sharp diffraction peaks, confirming their crystalline state. The identified characteristic peaks for each APIs at 2θ angles are as follows: EMPA—12.96°; 21.28°; 35.26°; DAPA—12.32°, 17.40°, 18.28°; Cur—9.12°, 14.64°, 17.40°; OA—8.56°; 13.76°; 16.38°. The results are in agreement with other reported data [7,34,35,36].

#### 3.1.4. The Encapsulation Efficiency (EE)

EE (%) is a parameter that indicates the proportion of APIs encapsulated in LPs, relative to the total amount of drugs used in the formulation process. It is well known that LPs are much more effective for encapsulating lipophilic drugs compared to hydrophilic ones [37]. Since the SGLT2 inhibitors selected for this study, DAPA and EMPA, are hydrophobic, we expect higher EE% values, due to their localization in the lipid bilayer where the alkyl “tails” of the fatty acids are located. The EE% value of DAPA/EMPA in LPs ranged from 57.51 ± 3.70% to 87.11 ± 3.00%, depending on the API:PC ratio. For EMPA, the highest EE%, 80.20 ± 5.41%, was recorded at an EMPA:PC ratio of 1:60, while in the case of DAPA, the highest EE%, 87.11 ± 3.00%, was obtained at a DAPA:PC ratio of 1:400 (Table 4). Regarding Cur, the maximum EE% value recorded was 75.40 ± 6.90%, corresponding to a Cur-PC ratio of 1:20. The EE% of DAPA and EMPA increased when they were co-loaded with Cur/OA, much more in the case of OA (Table 5).

The highest values were recorded for formulation (b), concerning the APIs (EMPA/DAPA-Cur/OA), with PC ratio of 1:60/1:20 for EMPA-Cur/OA and 1:400/1:20 for DAPA-Cur/OA. For EMPA, the EE% for formulation (b) was 84.20 ± 3.08% in PVP-EMPA-Cur-LPs, while for PVP-EMPA-OA-LPs, it was significantly higher at 96.20 ± 4.03%. In the case of DAPA, the recorded EE% was more than 95%, specifically 96.10 ± 2.71% (PVP-DAPA-Cur-LPs) and 97.40 ± 5.01% (PVP-DAPA-OA-LPs). The increased EE% of SGLT2 inhibitors (DAPA/EMPA) co-loaded with Cur/OA may be attributed to interactions between these molecules, possibly through non-covalent attachment of the functional groups of these APIs and their site in the lipid bilayer. It has been noted that the EE% of hydrophobic drugs in the liposomal matrix increases with a decrease in membrane fluidity [11]. Thus, in our study, the increase in EE% of DAPA/EMPA when co-loaded with OA, compared with Cur, could also be attributed to the effect of OA in reducing liposomal membrane fluidity.

### 3.2. Stability of LPs

#### 3.2.1. Storage

**Figure 4:**
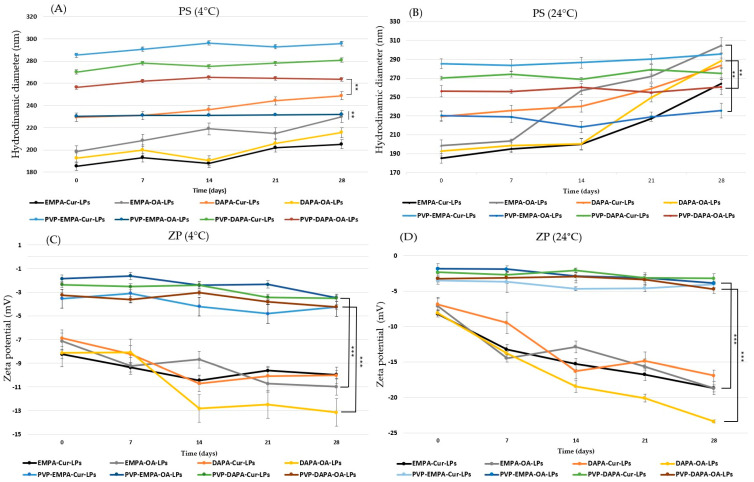
Physical parameters: (**A**) PS at 4 °C; (**B**) PS at 24 °C; (**C**) ZP at 4 °C; (**D**) ZP at 24 °C of coated/uncoated APIs-LPs storage during 28 days. The results are expressed as mean ± standard deviation, *n* = 3 of the obtained values. Statistical analysis: ** *p* < 0.01, *** *p* < 0.001.

The long-term storage stability of both uncoated and PVP-coated APIs-LPs was assessed in terms of PS and ZP, in the hydrated form of LPs, over a period of 4 weeks at two set temperatures, 24 °C and 4 °C. The analysis of the results (Figure 4) showed no significant changes in PS during storage at 4 °C. However, a slight increase rate was observed for APIs-OA-LPs, with growth rates of 18.70% for EMPA-OA-LPs and 11.83% for DAPA-OA-LPs (*p* < 0.001).

Under the same experimental conditions, PVA-APIs-LPs demonstrated significantly greater stability, with the highest growth rate observed for of PVA-DAPA-Cur-LPs, which was only 3.96% (*p* < 0.001). Similar results were observed during storage at 24 °C, with PVP-APIs-LPs exhibiting greater stability than their uncoated counterparts. The highest PS growth rates were recorded for EMPA-OA-LPs and DAPA-OA-LPs at 134.44% and 49.60%, respectively, while the growth for PVP-EMPA/DAPA-OA-LPs was approximately 2%. Our findings support the idea that the physical barrier formed by PVP on the lipid bilayer of LPs enhances storage stability at both 4 °C and 24 °C. This is achieved by reducing membrane fluidity, decreasing membrane fusion between particles, minimizing lipid oxidative degradation, and maintaining the structural integrity [27,33].

Another indicator of lipid particle stability is ZP, the negative value indicating repulsive forces and decreasing aggregation of particles, but during long-term storage and under certain temperature conditions, this physical parameter may undergo changes.

During the storage, a decrease in ZP was noted, especially at 24 °C, for APIs-LPs. A decreasing rate of 161.72% and 187.55% were recorded for EMPA-OA-LPs and DAPA-OA-LPs, respectively. In the case of PVP-APIs-LPs, insignificant decreases in ZP values were recorded, which again highlights the protective effect of the polymer, with a statistical difference between them (*p* < 0.001). A decrease in ZP may occur as a result of lipid membrane degradation, oxidation, or hydrolysis of PC, which leads to the release of anionic fatty acids that can accumulate on the surface of the particles [27].

#### 3.2.2. Osmotic Stress

**Figure 5:**
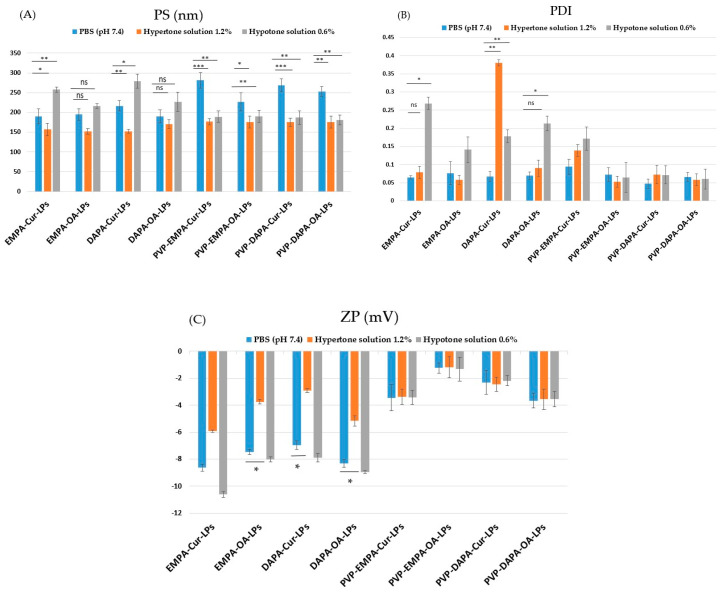
Physical parameters in different stress environments: (**A**) PS; (**B**) PDI; (**C**) ZP of APIs-LPs and PVP-APIs-LPs The results are expressed as mean ± standard deviation, *n* = 3 of the obtained values. Statistical analysis: * *p* < 0.05, ** *p* < 0.01, *** *p* < 0.001, ns: non-significant > 0.05.

In a hypertonic environment (NaCl 1.2%), LPs gradually dehydrate, resulting in a slight decrease in PS, while in a hypotonic environment (NaCl 0.6%), the LPs show a slight increase in size. Figure 5 illustrates the response of APIs-LPs and PVP-APIs-LPs to osmotic stress, in comparison to PBS at pH 7.4.

**Figure 6:**
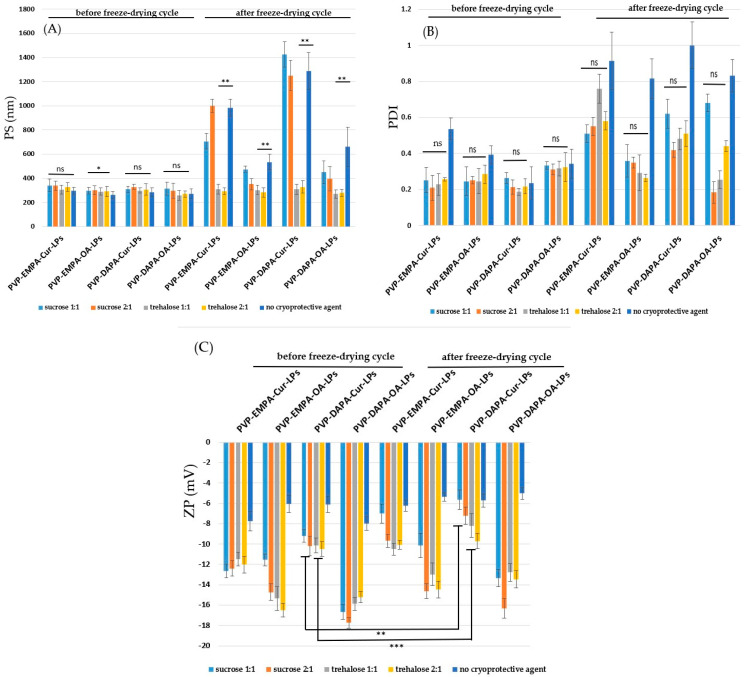
Physical parameters: (**A**) PS; (**B**) PDI; (**C**) ZPof sugar-coated PVP-APIs-LPs before and after freeze-drying. The results are expressed as mean ± standard deviation, *n* = 3 of the obtained values. Statistical analysis: * *p* < 0.05, ** *p* < 0.01, *** *p* < 0.001, ns: non-significant > 0.05.

As expected, under hypertonic conditions, the PS (Figure 5A) of APIs-LPs decreased, while it increased under hypotonic conditions compared to the values recorded in PBS at pH 7.4. In contrast, PVP-APIs-LPs showed no significant differences between hypertonic and hypotonic conditions, indicating the beneficial effect of PVP film on the stability of lipid particles. Regarding the PDI (Figure 5B), PVP-APIs-LPs exhibited no significant differences among PBS hypertonic and hypotonic solutions. For APIs-LPs, the hypertonic solution generally did not significantly affect the PDI (*p* > 0.05), except for DAPA-Cur-LPs, which were significantly affected (*p* < 0.01). Additionally, the hypotonic solution was found to increase the PDI of APIs-LPs. Concerning the ZP consistent with the literature, the presence of ions disturbed the surface charge balance of LPs, due to interactions between electrolytes and negative surface charges of the LPs [23,38]. Thus, in the hypertonic environment, the ZP values (Figure 6C) of APIs-LPs increased while in the hypotonic environment, they slightly decreased. The PVP coating APIs-LPs effectively kept the ZP stable at all salt concentrations.

#### 3.2.3. Freeze-Drying Stress

It has been reported that the freeze-drying process can have a negative impact on the lipid membrane, and coating it with cryoprotectants may provide a protective effect [18]. To evaluate the stability of PVP-APIs-LPs, two sugars—trehalose and sucrose—were selected, each tested in two different ratios relative to the lipid components (sugars: PC—1:1, 2:1). The physical parameters (PS, PDI, and ZP) of both sugar-coated and uncoated APIs-LPs were analyzed before and after freeze-drying (Figure 6). Regarding the PS, it was observed that, after freeze-drying, the size of all trehalose-coated PVP-APIs-LPs did not show a significant change.

This finding supports the positive effect of trehalose on the stability of lipid membranes, as reported in the literature [39]. In contrast, sucrose was less effective; an increase in PS was observed, particularly for PVP-EMPA/DAPA-Cur-LPs, which recorded PS values of 706.40 ± 95.20 and 1426.00 ± 106.50, respectively. These values were similar to those of the uncoated PVP-EMPA/DAPA-LPs [18,40].

The precise cryoprotective mechanism is not fully understood. However, the literature suggests that these disaccharides coat the lipid surface by forming hydrogen bonds with the carbonyl groups of PVP. This interaction creates a glassy matrix upon freezing, which increases viscosity and reduces molecular mobility [39]. Consequently, this helps prevent the aggregation of vesicles during the freeze-drying process. The protective effect of sugars is further supported by the PDI values. For all sugar-coated PVP-APIs-LPs, the PDI values after freeze-drying were lower than those of the uncoated LPs. However, for the sugar-coated EMPA/DAPA-Cur-LPs, the recorded PDI values were higher than those of the corresponding formulations recorded before freeze-drying. Lastly, the positive impact of both trehalose and sucrose on lipid membrane stability is reinforced by the ZP values. The ZP values recorded before and after freeze-drying were lower (more negative) for the sugar-coated formulations compared to the uncoated ones. Changes in the ZP of LPs are thought to result from membrane destabilization and drug leakage through the lipid bilayer during phase transitions, according to Trenkenschuh et al. [39], showing that when the cryoprotective effect is less effective, the amount of residual water on the surface of the LPs increases. This increase in water content raises the phase transition temperature (Tm) of the lipid bilayer, which corresponds to stronger van der Waals forces between the phospholipids.

### 3.3. Thermal Analysis

#### 3.3.1. Thermogravimetry (TG)/Differential Thermal Analysis (DTA)

**Figure 7:**
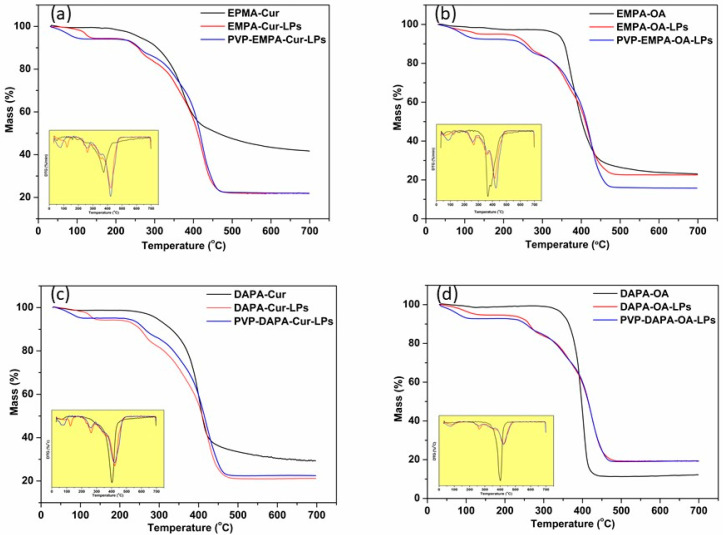
TG and DTG curves of: (**a**) EMPA-Cur based LPs and physical mixture; (**b**) EMPA-OA based LPs and physical mixture; (**c**) DAPA-Cur based LPs and physical mixture; (**d**) DAPA-OA based LPs and physical mixture.

Thermogravimetric analysis was performed to evaluate the thermal stability of APIs-LPs and PVP-APIs-LPs in comparison to pure APIs. The thermograms (TG/DTA and DTG) for the pure APIs (EMPA, DAPA, Cur, OA) are shown in SI (Figure S1–S4). All profiles displayed similar behavior, characterized by a significant degradation phase, with weight changes associated with the disintegration process occurring between 300 °C and 500 °C. The recorded onset (T_onset_) and endset (T_endset_) temperature values for the APIs were as follows: EMPA 323.4 °C/444.4 °C (Figure S1), DAPA 394.9 °C/417.0 °C (Figure S2), Cur 321.0 °C/412.2 °C (Figure S3), and OA 392.8 °C/426.6 °C (Figure S4). For the physical mixture of APIs (EMPA/DAPA-Cur/OA), a decrease in both T_onset_ and T_endset_ were observed, indicating a physical interaction between APIs (Figure 7). The T_onset/endset_ for EMPA-Cur/OA were recorded at 352.6 °C/417.7 °C and 330.8 °C/402.6 °C, respectively (Figure 7a,b). Similar decreases were noted for DAPA-Cur/OA, with T_onset/endset_ values of 378.2 °C/413.6 °C and 367.0 °C/420.9 °C, respectively (Figure 7c,d).

In the case of APIs-LPs, the degradation process was multi-step, with T_onset_ values increasing compared to those of the pure APIs and their physical mixture. The first step occurred between 50 °C and 200 °C, showing a slight weight loss, attributed to the loss of residual moisture and to volatile compounds. The second stage took place between 250 and 350 °C, during which an 11% mass loss was recorded, attributed to the starting of the degradation process. The most substantial mass loss occurred in the third stage, with DTG peak at 423.7 °C (EMPA-Cur-LPs), 417.3 °C (EMPA-OA-LPs), 421.6 °C (DAPA-Cur-LPs), and 425.4 °C (DAPA-OA-LPs) (Figure 7).

The covering of APIs-LPs with the amphiphilic polymer PVP had a limited effect on the thermal behavior of APIs-LPs, as the thermograms remained quite similar, with only a slight increase in T_endset_ observed. Moreover, when the temperature increased to over 350 °C, a considerable weight loss was recorded, as with other studies reported [17]. The total weight loss at 700 °C for PVP-coated and uncoated APIs-LPs was comparable, with slight differences in T_endset_ values, as follows: PVP-EMPA-Cur/EMPA-Cur: 448.8 °C/447.1 °C; PVP-EMPA-OA/ EMPA-OA: 449.6 °C/445.0 °C; PVP-DAPA-Cur/DAPA-Cur: 451.4 °C/446.2 °C; PVP-DAPA-OA/ DAPA-OA: 450.0 °C/449.6 °C.

#### 3.3.2. Differential Scanning Calorimetry (DSC)

**Figure 8:**
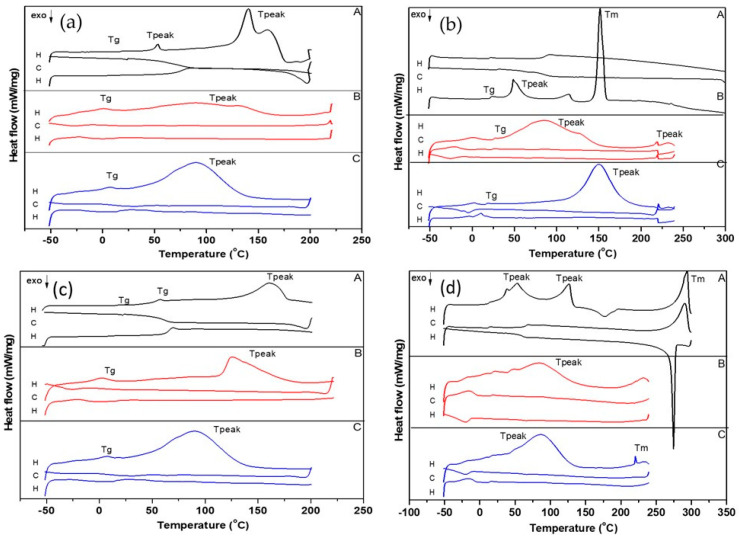
DSC curves of: (**a**) EMPA-Cur based LPs and physical mixture: (A) EMPA-Cur, (B) EMPA-Cur-LPs, (C) PVP-EMPA-Cur-LPs; (**b**) EMPA-OA based LPs and physical mixture: (A) EMPA-OA, (B) EMPA-OA-LPs, (C) PVP-EMPA-OA-LPs; (**c**) DAPA-Cur based LPs and physical mixture: (A) DAPA-Cur, (B) DAPA-Cur-LPs, (C) PVP-DAPA-Cur-LPs; (**d**) DAPA-OA based LPs and physical mixture: (A) DAPA-OA, (B) DAPA-OA-LPs, (C) PVP-DAPA-OA-LPs. H—heating cycle; C—cooling cycle; ↓—exothermic process.

The DSC analysis completes the thermal analysis and was conducted to investigate the crystallinity and the melting point of APIs as well as their corresponding LPs (PVP-APIs-LPs, APIs-LPs). The DSC thermograms display exothermic/endothermic peaks, which are reported in SI (Table S1, Figures S5–S8) as follows: T_g_ (glass transition temperature—T_onset_/T_midpoint_); T_peak_ (temperature at maximum peak height); T_m_ (melting point); and T_c_ (cold crystallization point). The details of the exothermic and endothermic peaks for the selected cycles, H (heating cycle, from −50 °C to 250 °C) and C (cooling cycle, from 250 °C to −50 °C), for pure APIs as well as for LPs formulations are shown in SI (Table S1). For Cur, Heffernan et al. [41] reported a T_m_ of 182.17 °C, while Jambhrunkar et al. [42] noted an endothermic point at 176 °C. In our study, an endothermic point at 180.1 °C was identified (Table S2, Figure S7), which is close to the reported values. The T_m_ of EMPA was observed at 153.2 °C (Table S1, Figure S5), consistent with the report of Ma et al. [43], while for DAPA, two endothermic peaks were identified at 48.1 °C and 64.5 °C (Table S2, Figure S6), in contrast to a single peak at 88.3 °C reported by Hu et al. [44]. For OA, a T_g_ was observed at 7.5 °C, followed by two endothermic peaks at 36.2 °C and 125.5 °C, corresponding to different transitions, and a T_c_ at 193 °C. The endothermic and exothermic effects may be influenced by trace amounts of water [45]. Various T_m_ over 270 °C have been reported in the literature for OA. In our study, the Tm of OA was identified at 315.0 °C, which coincides with its decomposition (Table S1, Figure S8). Figure 8a–d presents the DSC thermograms for the physical mixtures of APIs and their corresponding LPs (APIs-LPs and PVP-APIs-LPs). The DSC curve for DAPA-OA exhibited multiple endothermic T_peaks_ and T_m_ at 294 °C, while DAPA-OA-LPs showed shifted endothermic T_peaks_ (48.2 °C and 84 °C), attributed to the incorporation of DAPA and OA into the lipid matrix. The increased T_peak_ value observed for PVP-DAPA-OA-LPs (86.9 °C) compared to the value recorded for DAPA-OA-LPs (84 °C) demonstrates that PVP provides thermal protection (Figure 8d). This additional thermal protective effect observed with PVP coating may be attributed to the thermodynamic stability of the polymer, which helps prevent the disruption of PC chains within the liposomal matrix. Furthermore, the steric hindrance provided by PVP contributes to enhanced particle stability under thermal stress [46].

The DSC curve of DAPA-Cur showed an endothermic T_peak_ at 160 °C, whereas for DAPA-Cur-LPs and PVP-DAPA-Cur-LPs, the corresponding T_peaks_ shifted to 125.8 °C and 89.9 °C, respectively (Figure 8c). As shown in Figure 8b, EMPA-OA undergoes multiple phase changes during temperature rise, with a maximum endothermic T_m_ of 151.4 °C. In the case of EMPA-OA-LPs, a broad peak is observed between 50 °C and 130 °C. With PVP surface modification (PVP-EMPA-OA-LPs), the endothermic T_peak_ shifts to 150.3 °C. For these LPs, the influence of EMPA is stronger than in other samples, as reflected in the T_peak_, which is close to the T_m_ of EMPA (153.2 °C). A similar behavior was observed for EMPA-Cur-LPs. While a strong T_peak_ was not detected for EMPA-Cur-LPs, a shifted endothermic T_peak_ of 86.5 °C was noted in the PVP-EMPA-Cur-LPs (Figure 8a). Our study highlighted that significant exothermic and endothermic effects occur during the first heating cycle. This indicates that the microcrystalline and physical properties of the samples change after this initial cycle, resulting in only minor thermal phenomena during subsequent cooling and heating cycles. Nonetheless, the thermal stability of the LPs remains unaffected up to 250 °C.

More thermogravimetric data, including the main degradation steps, as well as the weight loss (%) are presented in Table S2.

### 3.4. In Vitro Release Study

**Figure 9:**
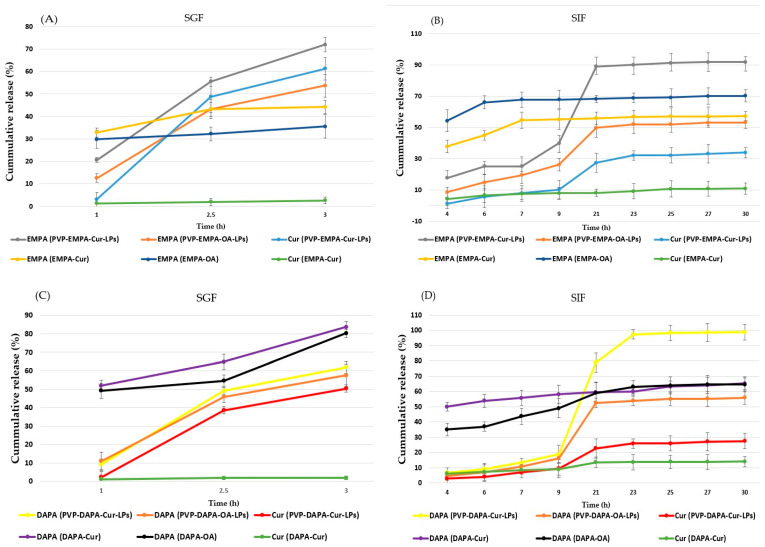
Cumulative release profile (%) of APIs (EMPA, DAPA, Cur) from PVP-APIs-LPs and API mixtures: (**A**) EMPA and Cur from PVP-EMPA-Cur/OA-LPs and EMPA-Cur/OA in SGF; (**B**) EMPA and Cur from PVP-EMPA-Cur/OA-LPs and EMPA-Cur/OA in SIF; (**C**) DAPA and Cur from PVP-DAPA-Cur/OA-LPs and DAPA-Cur/OA in SGF; (**D**) DAPA and Cur from PVP-DAPA-Cur/OA-LPs and DAPA-Cur/OA in SIF.

The in vitro release profile of PVP-APIs-LPs, compared to the physical API mixture, was evaluated using different simulated fluids (SGF, SIF) over various time points, as shown in Figure 9. Based on the hydrophobic nature of the LP matrix, it is expected that the API release from the LP formulation would be slower than from the physical API mixture. Additionally, the presence of OA is anticipated to enhance this slower release due to its structural similarity to Col. OA is also known to improve the stability of the liposomal matrix, resulting in a more delayed and sustained drug release [13,47,48].

The release profiles of DAPA and Cur from PVP-DAPA-Cur/OA in SGF were similar. At 3 h, the release rate of DAPA was 61.86 ± 6.02% for PVP-DAPA-Cur-LPs and 57.56 ± 3.16% for PVP-DAPA-OA-LPs (Figure 9C). This slight difference in release rate could be attributed to OA’s effect of slightly delaying the drug release.

Regarding the release of EMPA from the EMPA-OA/Cur mixture, it reached a peak of 80.29 ± 2.95% and 83.72 ± 2.25%, respectively, with a burst release occurring at 1 h. The release of Cur from the PVP-DAPA-Cur-LPs reached 50.44 ± 2.01% at 3 h, exhibiting a slow, sustained release. In contrast, the Cur release from the DAPA-Cur mixture was significantly lower, at only 1.93% ± 0.9 (*p* < 0.001). These results suggest that the liposomal formulation enhanced the release profile of Cur. This improvement can also be attributed to the more water-soluble, amorphous form of Cur in the LPs, compared to the less soluble crystalline form found in the DAPA-Cur mixture. On the other hand, PVP enhances the stability of the liposomal matrix by increasing resistance to low pH and pepsin, thereby prolonging the drug release profile in the stomach [49]. A slow release in the gastric environment is beneficial for oral delivery systems, as it allows a greater amount of APIs to be available for absorption in the intestine [49,50].

In SIF, DAPA release from the DAPA-Cur/OA mixture reached 49 ± 5.90% and 58.09 ± 6.30%, respectively, at 9 h. Afterward, a slight increase was observed, with release reaching 64.61 ± 5.80% and 65.80 ± 6.80% at the end of the experiment (30 h). In contrast, DAPA release from PVP-DAPA-Cur/OA-LPs was slower and more sustained. At 9 h, the release was lower, at 16.3 ± 5.08% and 18.93 ± 3.09%, respectively. However, it steadily increased to 55.98 ± 5.91% and 98.73 ± 4.03% by 30 h (Figure 9D). The statistically significant difference (*p* < 0.001) in DAPA release between PVP-DAPA-OA-LPs and PVP-DAPA-Cur-LPs is likely due to the presence of OA, which enhances the stability of the liposomal matrix, similar to its effect in SGF.

The release of Cur from the DAPA-Cur in SIF exhibited a rapid release pattern, but with a very low percentage released within the first 4 h (6.23 ± 1.33%), and it remained below 14.05 ± 3.5% at the end of the experiment. As observed in SGF, this behavior can be attributed to the poor solubility of crystalline Cur in SIF. In contrast, PVP-DAPA-Cur-LPs displayed a sustained and increasing release of Cur, likely due to the transition of Cur from its crystalline to amorphous form, which is more soluble in aqueous environments [18]. As shown in Figure 9A,B, the release behavior of EMPA and Cur from PVP-EMPA-Cur/OA LPs exhibited a sustained and progressively increasing release compared to the EMPA-Cur/OA mixture, particularly in SIF. At 9 h, the release of EMPA from PVP-EMPA-Cur/OA was 26.15 ± 4.08% and 39.93 ± 5.09%, respectively. At the end of the experiment, the recorded values were 53.21 ± 7.90% for PVP-EMPA-OA-LPs, as well as significantly higher values of 91.9 ± 7.31% for PVP-EMPA-Cur-LPs. The in vitro release findings suggest that the LPs formulations provide a sustained and delayed release of APIs.

## 4. Conclusions

In this study, LPs containing SGLT2 inhibitors (EMPA, DAPA) and Cur were formulated as novel synergistic strategies for HF. The PS is closely related to ratio of lipid components (PC:Col) and APIs to lipid components, as well as extrusion process, PVP coating, and adding of OA, to enhance the stability of liposomal membrane. The PS decreased up to 170 nm with extrusion process, and all formulations showed negative ZP, confirming good colloidal stability. STEM confirmed stable, spherical LPs with slight size increase and surface roughness after PVP coating. The amorphous state of APIs in LPs, indicating successful incorporation and interaction with the lipid matrix, was confirmed through XRD analysis. The EE% of SGLT2 inhibitors (DAPA, EMPA) exceeded 80%, especially when co-loaded with OA, with the highest value of 96.20 ± 4.03% recorded for PVP-EMPA-OA-LPs. PVP coating significantly enhances the long-term storage stability of APIs-LPs, especially at 24 °C, as well as resistance to osmotic stress. In addition, the stability of APIs-LPs to freeze-drying stress was increased by trehalose, used as cryoprotectant. The thermal stability of APIs increased by incorporation into the liposomal matrix, and PVP coating provided additional thermal protection; all formulations remained stable up to 250 °C. In vitro release studies demonstrated that PVP-coated liposomal formulations enable sustained and delayed EMPA/DAPA release with enhanced control observed in the presence of OA and Cur. All these findings are strong evidence to continue our research with in vivo tests to prove their effectiveness in HF, using animal models.

## Figures and Tables

**Table 1:** The formulation of blank, single-loaded, and co-loaded APIs-LPs.

LPs		Extrusion	Col (mg)	PC (mg)	API:PC (*w*/*w*)	API (mg)
LPs@1a		-	5	30	-	-
LPs@2a		-	5	20	-	-
LPs@1b		✓	5	30	-	-
LPs@2b		✓	5	20	-	-
Cur-LPs@1		✓	5	20	1:10	2
Cur-LPs@2		✓	5	30	1:15	2
OA-LPs@1		✓	5	20	1:10	2
OA-LPs@2		✓	5	30	1:15	2
EMPA-LPs@1		✓	5	20	1:25	0.800
EMPA-LPs@2		✓	5	30	2:75	0.800
DAPA-LPs@1		✓	5	20	1:100	0.200
DAPA-LPs@2		✓	5	30	1:150	0.200
EMPA-Cur-LPs(a)	EMPA	✓	5	20	1:30	0.667
	Cur	✓	5	20	1:10	2
EMPA-Cur-LPs(b)	EMPA	✓	5	20	1:60	0.333
	Cur	✓	5	20	1:20	1
EMPA-Cur-LPs(c)	EMPA	✓	5	20	1:75	0.266
	Cur	✓	5	20	1:25	0.800
EMPA-Cur-LPs(d)	EMPA	✓	5	20	1:90	0.222
	Cur	✓	5	20	1:30	0.666
EMPA-OA-LPs(a)	EMPA	✓	5	20	1:30	0.666
	OA	✓	5	20	1:10	2
EMPA-OA-LPs(b)	EMPA	✓	5	20	1:60	0.333
	OA	✓	5	20	1:20	1
EMPA-OA-LPs(c)	EMPA	✓	5	20	1:90	0.220
	OA	✓	5	20	1:30	0.666
EMPA-OA-LPs(d)	EMPA	✓	5	20	1:100	0.200
	OA	✓	5	20	1:35	0.571
DAPA-Cur-LPs(a)	DAPA	✓	5	20	1:200	0.100
	Cur	✓	5	20	1:10	2
DAPA-Cur-LPs(b)	DAPA	✓	5	20	1:400	0.050
	Cur	✓	5	20	1:20	1
DAPA-Cur-LPs(c)	DAPA	✓	5	20	1:500	0.040
	Cur	✓	5	20	1:22	0.900
DAPA-Cur-LPs(d)	DAPA	✓	5	20	1:500	0.040
	Cur	✓	5	20	1:25	0.800
DAPA-OA-LPs(a)	DAPA	✓	5	20	1:200	0.100
	OA	✓	5	20	1:10	2
DAPA-OA-LPs(b)	DAPA	✓	5	20	1:400	0.050
	OA	✓	5	20	1:20	1
DAPA-OA-LPs(c)	DAPA	✓	5	20	1:500	0.040
	OA	✓	5	20	1:25	0.800
DAPA-OA-LPs(d)	DAPA	✓	5	20	1:600	0.033
	OA	✓	5	20	1:30	0.666

**Table 2:** The physical parameters of LPs and single APIs-LPs.

LPs/ APIs-LPs	PS (nm)	PDI	ZP (mV)
LPs@1a	1796.00 ± 0.22 ***	0.916 ± 0.017 ^ns^	−31.79 ± 0.57 **
LPs@2a	871.80 ± 0.15 ***	0.745 ± 0.015 ^ns^	−26.41 ± 0.51 **
LPs@1b	199.60 ± 2.57 ***	0.351 ± 0.019 ^ns^	−16.29 ± 0.82 **
LPs@2b	191.60 ± 1.85 ***	0.484 ± 0.015 ^ns^	−14.86 ± 0.34 **
Cur-LPs@1	166.30 ± 4.70 ^ns^	0.488 ± 0.014 ^ns^	−12.28 ± 4.47 ^ns^
Cur-LPs@2	179.40 ± 5.30 ^ns^	0.045 ± 0.011 **	−13.40 ± 4.09 ^ns^
OA-LPs@1	221.20 ± 4.30 ^ns^	0.321 ± 0.011 ^ns^	−20.31 ± 3.12 ^ns^
OA-LPs@2	236.80 ± 3.20 ^ns^	0.244 ± 0.015 ^ns^	−13.68 ± 1.75 ^ns^
EMPA-LPs@1	190.50 ± 12.30 ^ns^	0.080 ± 0.066 **	−15.27 ± 3.89 ^ns^
EMPA-LPs@2	198.90 ± 2.60 ^ns^	0.010 ± 0.057 **	−16.27 ± 1.25 ^ns^
DAPA-LPs@1	178.40 ± 4.00 ^ns^	0.062 ± 0.028 **	−10.54 ± 1.05 ^ns^
DAPA-LPs@2	199.10 ± 5.60 ^ns^	0.236 ± 0.031 ^ns^	−16.10 ± 2.03 ^ns^

The results are expressed as mean ± standard deviation, *n* = 3 of the obtained values. Statistical analysis: ** *p* < 0.01, *** *p* < 0.001, ns: non-significant > 0.05 between the extruded and un-extruded LPs, with the same PC:Col ratio, and also between extruded single APIs-LPs and extruded LPs.

**Table 3:** The physical parameters of co-loaded APs-LPs/PVP-APIs-LPs.

LPs	PS (nm)	PDI	ZP (mV)
EMPA-Cur-LPs(a)	164.20 ± 1.21	0.031 ± 0.01	−8.10 ± 1.67
EMPA-Cur-LPs(b)	206.00 ± 12.30	0.082 ± 0.01	−10.54 ± 2.05
EMPA-Cur-LPs(c)	166.50 ± 3.11	0.122 ± 0.03	−10.42 ± 1.53
EMPA-Cur-LPs(d)	194.90 ± 7.32	0.147 ± 0.01	−10.07 ± 2.09
EMPA-OA-LPs(a)	200.90 ± 3.91	0.065 ± 0.02	−7.46 ± 2.61
EMPA-OA-LPs(b)	203.40 ± 2.01	0.191 ± 0.01	−7.49 ± 1.53
EMPA-OA-LPs(c)	177.50 ± 2.21	0.056 ± 0.01	−8.92 ± 2.01
EMPA-OA-LPs(d)	178.40 ± 3.22	0.072 ± 0.06	−9.15 ± 2.06
DAPA-Cur-LPs(a)	226.30 ± 5.11	0.183 ± 0.11	−7.43 ± 0.47
DAPA-Cur-LPs(b)	225.90 ± 10.23	0.070 ± 0.01	−6.95 ± 0.29
DAPA-Cur-LPs(c)	212.90 ± 3.91	0.164 ± 0.01	−7.04 ± 0.81
DAPA-Cur-LPs(d)	235.10 ± 5.31	0.141 ± 0.11	−8.88 ± 0.96
DAPA-OA-LPs(a)	198.10 ± 3.72	0.147 ± 0.10	−7.48 ± 0.94
DAPA-OA-LPs(b)	182.00 ± 2.71	0.047± 0.21	−10.44 ± 0.68
DAPA-OA-LPs(c)	205.90 ± 6.92	0.049 ± 0.13	−9.72 ± 1.05
DAPA-OA-LPs(d)	197.00 ± 8.01	0.047 ± 0.22	−8.93 ± 1.08
PVP-EMPA-Cur-LPs(b)	289.80 ± 2.01 **	0.147 ± 0.07 ^ns^	−6.97 ± 1.49 *
PVP-EMPA-OA-LPs(b)	236.2 ± 7.31 ^ns^	0.084 ± 0.05 **	−5.79 ± 2.14 *
PVP-DAPA-Cur-LPs(b)	271.6 ± 3.11 *	0.051 ± 0.01 ^ns^	−5.60 ± 1.34 ^ns^
PVP-DAPA-OA-LPs(b)	264.1 ± 2.20 *	0.055 ± 0.01 ^ns^	−7.61 ± 1.52 *

The results are expressed as mean ± standard deviation, *n* = 3 of the obtained values. Statistical analysis: * *p* < 0.05, ** *p* < 0.01, ns: non-significant > 0.05 between the PVP-APIs-LPs and the corresponding uncoated APIs-LPs.

**Table 4:** The encapsulation efficiency (%) of APIs (EMPA, DAPA, Cur) in API-LPs.

API-LPs	API:PC Ratio (*w*/*w*)									
	1:10	1:20	1:30	1:60	1:90	1:105	1:200	1:400	1:500	1:540
EMPA-LPs	-	-	69.14 ± 8.20	80.20 ± 5.41 ^ns^	65.01 ± 4.30 ^ns^	57.51 ± 3.70 ^ns^	-	-	-	-
DAPA-LPs	-	-	-	-	-	-	81.51 ± 6.03	87.11 ± 3.00 ^ns^	72.09 ± 5.19 ^ns^	69.12 ± 7.60 ^ns^
Cur-LPs	58.32 ± 5.02	75.40 ± 6.90 ^ns^	67.42 ± 5.14 ^ns^	48.60 ± 4.07 ^ns^	-	-	-	-	-	-

The results are expressed as mean ± standard deviation, *n* = 3 of the obtained values. Statistical analysis: ns: non-significant > 0.05 between different API:PC ratio of APIs-LPs.

**Table 5:** The encapsulation efficiency (%) of APIs (DAPA, EMPA, Cur) in PVP-APIs-LPs.

LPs	PVP-EMPA-Cur-LPs		PVP-EMPA-OA-LPs	PVP-DAPA-Cur-LPs		PVP-DAPA-OA-LPs
	EMPA	Cur	EMPA	DAPA	Cur	DAPA
(a)	71.00 ± 2.03	60.30 ± 3.06	76.00 ± 1.03	82.00 ± 2.09	65.30± 4.02	86.50 ± 4.92
(b)	84.20 ± 3.08	75.60 ± 6.62 ^ns^	96.20 ± 4.03 ^ns^	96.10 ± 2.71	80.10± 3.09 ^ns^	97.40 ± 5.01 ^ns^
(c)	70.00 ± 1.06	67.90 ± 5.02 ^ns^	86.20 ± 1.09 ^ns^	85.20 ± 1.89	74.90± 4.92 ^ns^	88.00 ± 7.02 ^ns^
(d)	65.20 ± 6.01	65.20 ± 3.05 ^ns^	84.00 ± 1.54 ^ns^	75.40 ± 2.06	51.00± 4.41 ^ns^	82.20 ± 4.41 ^ns^

(a), (b), (c), and (d) denote the four formulations for each PVP-APIs-LPs (EMPA/DAPA-Cur/OA-LPs) presented in Table 1. The results are expressed as mean ± standard deviation, *n* = 3 of the obtained values. Statistical analysis: ns: non-significant > 0.05 between the co-loaded PVP-APIs-LPs (a), (b), (c), and (d).

## Data Availability

The data could be requested from the authors.

## Supplementary Materials

The following supporting information can be downloaded at: https://www.mdpi.com/article/10.3390/pharmaceutics17080969/s1. Figure S1. TG, DTA and DTG curves of EMPA pure substance. Figure S2. TG, DTA and DTG curves of DAPA pure substance. Figure S3. TG, DTA and DTG curves of Cur pure substance. Figure S4. TG, DTA and DTG curves of OA pure substance. Figure S5. DSC curves of EMPA pure substance. Figure S6. DSC curves of DAPA pure substance. Figure S7. DSC curves of Cur pure substance. Figure S8. DSC curves of OA pure substance. Table S1. The results of differential scanning calorimetry (DSC) analysis: glass transition (Tg), melting (Tm), and crystallization (Tc) temperatures of APIs and its liposomal formulations. Table S2. Thermogravimetric data comprising the main degradation steps, as well as weight loss (%) and the amount of the analyzed samples (mg).
