# Supplementary material for: SGLT2 Inhibitors and Curcumin Co-loaded Liposomal Formulations as Synergistic Delivery Systems for Heart Failure Therapy
Source: Pharmaceutics. 2025 Jul 26;17(8):969. doi: 10.3390/pharmaceutics17080969 (PMC12389357; doi:10.3390/pharmaceutics17080969)
Supplement: Supplementary file 1 [file pharmaceutics-17-00969-s001.zip › pharmaceutics-3750948-supplementary.pdf]

Supplementary Information (SI)

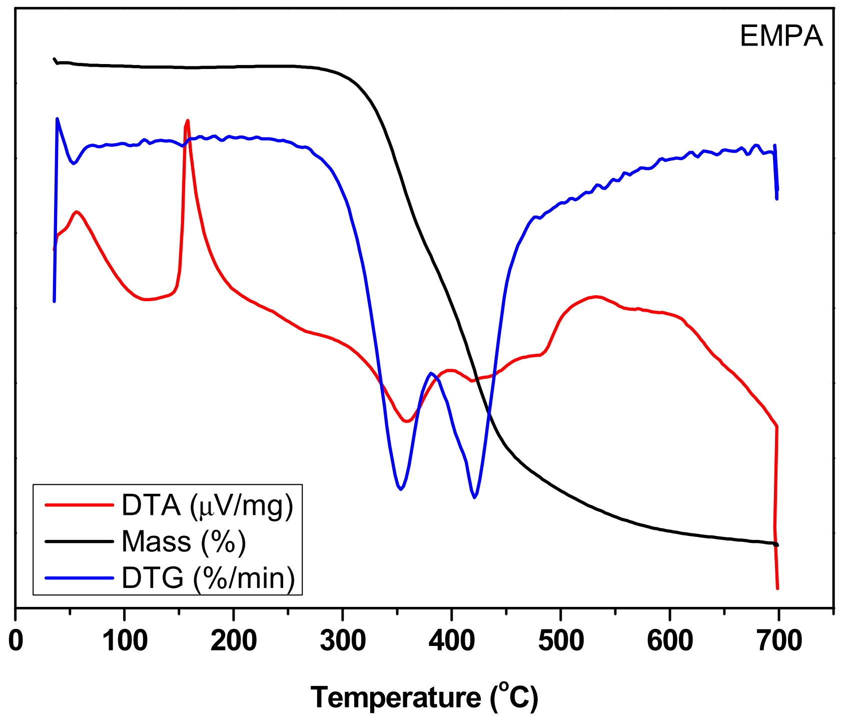

Figure S1. TG, DTA and DTG curves of EMPA pure substance.

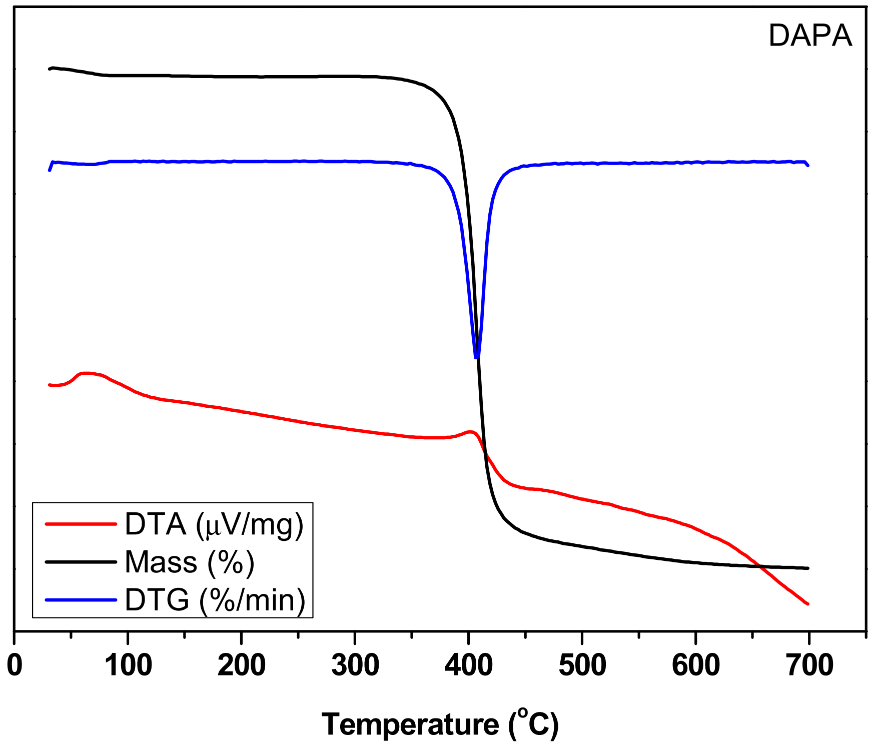

Figure S2. TG, DTA and DTG curves of DAPA pure substance.

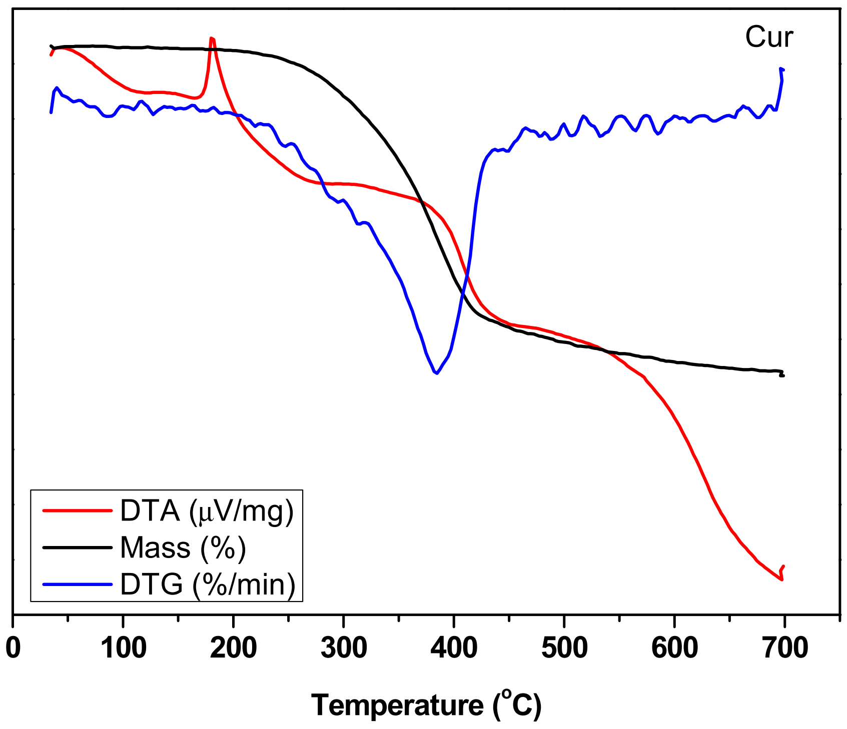

Figure S3. TG, DTA and DTG curves of Cur pure substance.

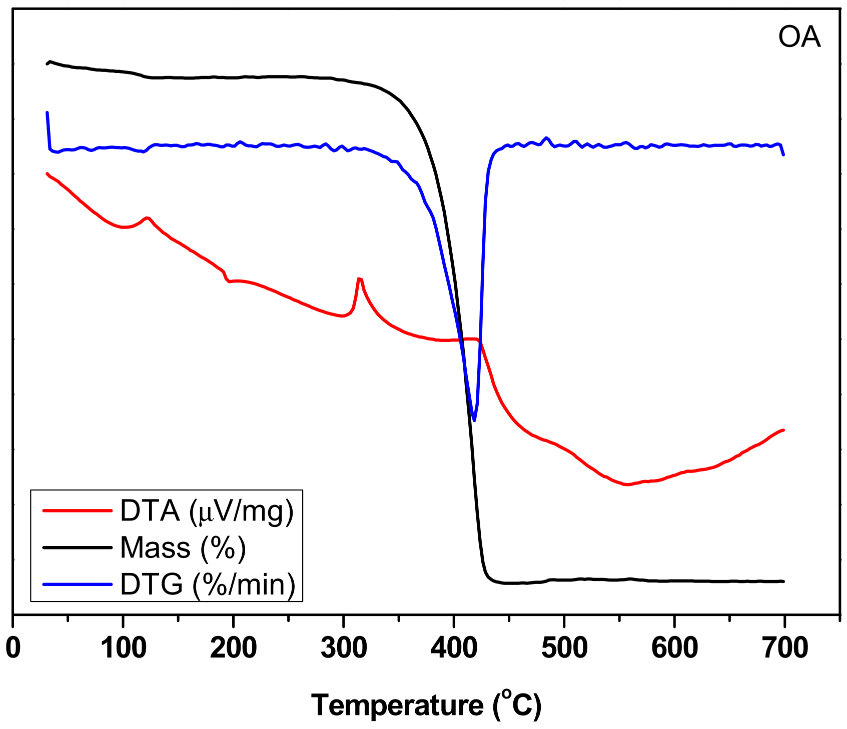

Figure S4. TG, DTA and DTG curves of OA pure substance.

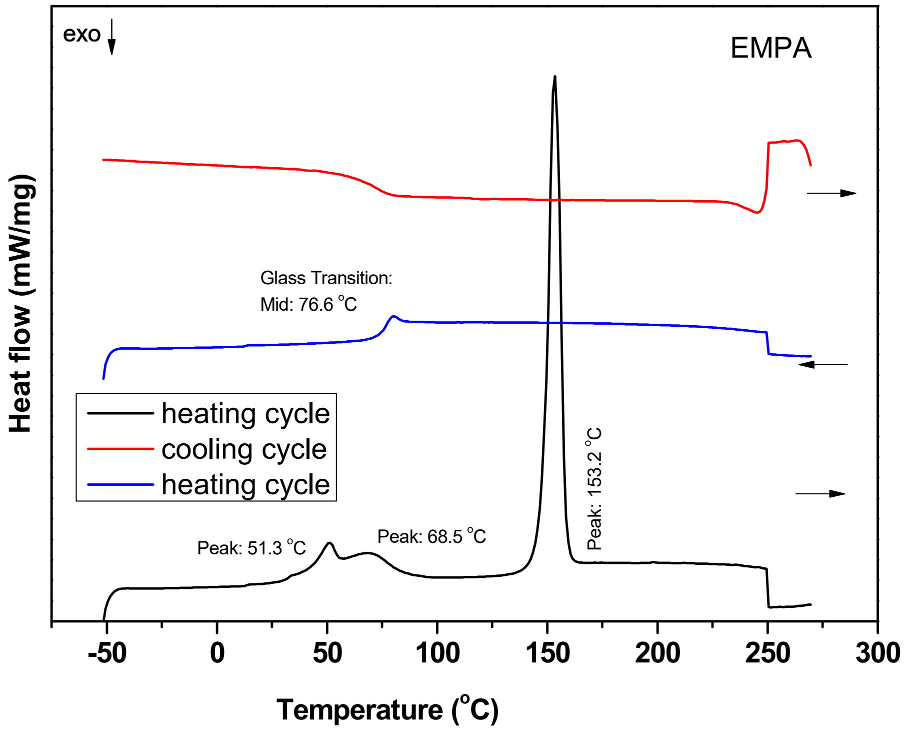

Figure S5. DSC curves of EMPA pure substance.

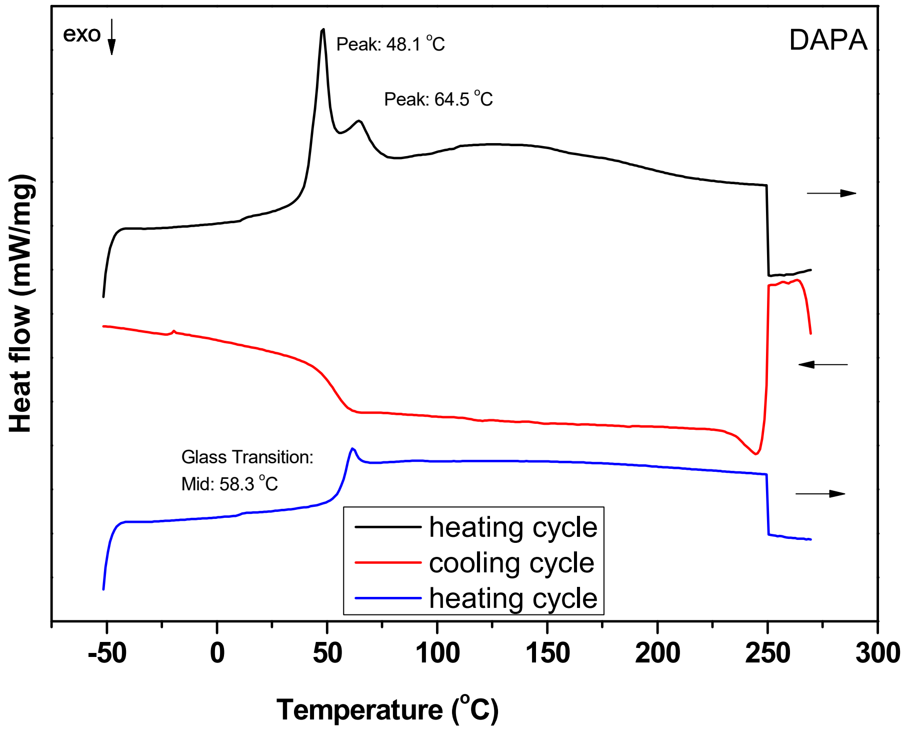

Figure S6. DSC curves of DAPA pure substance.

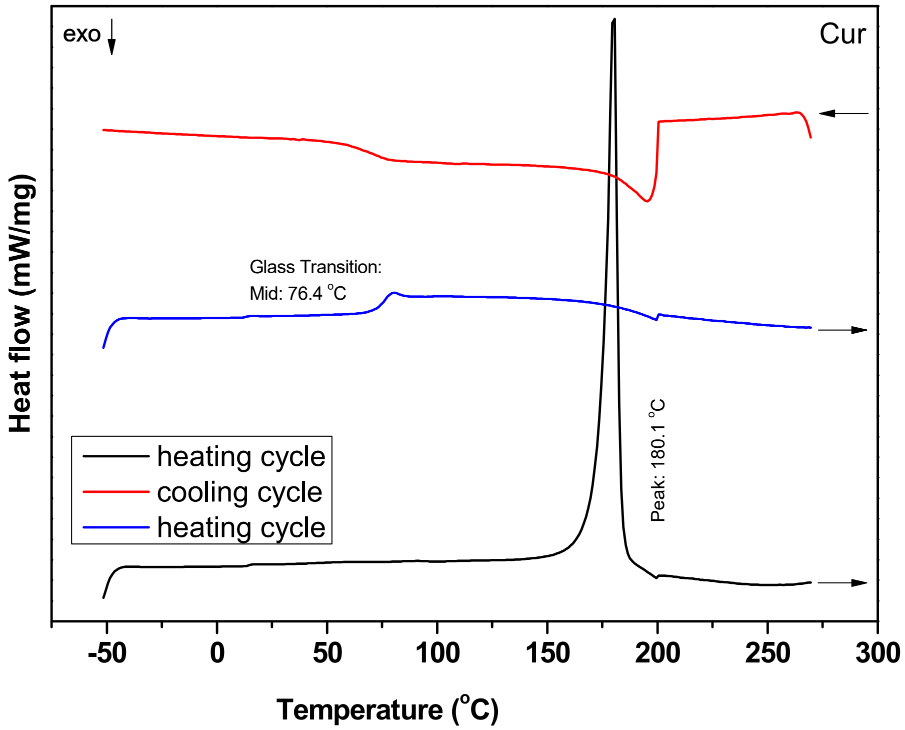

Figure S7. DSC curves of Cur pure substance.

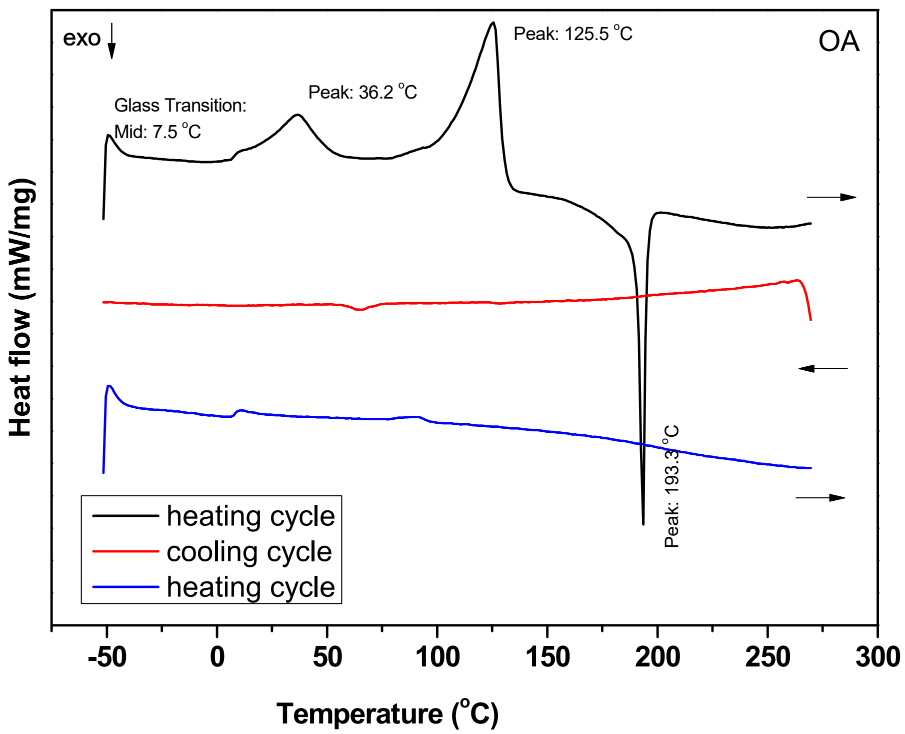

Figure S8. DSC curves of OA pure substance.

Table S1. The results of differential scanning calorimetry (DSC) analysis: glass transition (Tg), melting (Tm), and crystallization (Tc) temperatures of APIs and its liposomal formulations.

Fluid	Mass probe (mg)	First heating cycle (°C)	Second heating cycle (°C)
EMPA	9.4	T peak = 51.3 (endo)<br>T peak = 68.5 (endo)<br>T m = 153.2 (endo)	T g = 74.2/76.6 (Onset/Mid)
DAPA	11.8	T peak = 48.1 (endo)<br>T peak = 64.5 (endo)<br>T peak = 100.1/150.0 (endo-broad peak)	T g = 56.6/58.3 (Onset/Mid)<br>T g = 56.6/58.3 (Onset/Mid)
Cur	7.8	T m = 180.1 (endo)	T g = 72.9/76.4 (Onset/Mid)
OA	2.2	T g = 7.0/7.5 (Onset/Mid)<br>T peak = 36.2 (endo)<br>T peak = 125.5 (endo)<br>T c = 193.3 (exo)	T g = 6.0/8.3 (Onset/Mid)
EMPA-Cur	6.1	T g = 17.2/17.8 (Onset/Mid)<br>T peak = 53.2 (endo)<br>T g = 140.6/158.9 (Onset/Mid)	T g = 16.7/17.1 (Onset/Mid)<br>T g = 27.1 /79.8 (Onset/Mid)
EMPA-Cur-LPs	7.2	T peak = 0.2 (endo)<br>T peak = 90.2 (endo)<br>T peak = 130.5 (endo)<br>T g = 140.6/158.9 (Onset/Mid)	T peak = -22.9 (endo)<br>T g = 10.4/11.7 (Onset/Mid)
PVP-EMPA-Cur-Lps	3.4	T g = 11.9/14.8 (Onset/Mid)<br>T peak = 86.5 (endo)	T peak = -22.6 (endo)<br>T g = 13.5/14.6 (Onset/Mid)
EMPA-OA	2.8	T g = 20.8/21.6 (Onset/Mid)<br>T peak = 48.5 (endo)<br>T g = 104.2/105.9 (Onset/Mid)<br>T m = 151.4 (endo)	T g = 19.3/20.2 (Onset/Mid)<br>T g = 84.9/88.1 (Onset/Mid)
EMPA-OA-LPs	4.8	T peak = 1.3 (endo)<br>T g = 24.8/25.5 (Onset/Mid)<br>T peak = 86.0 (endo)<br>T peak = 130.0 (endo)	T peak = -21.4 (endo)<br>T g = 23.3/24.2 (Onset/Mid)
PVP-EMPA-OA-LPs	5.2	T peak = 2.6 (endo)<br>T g = 15.9/17.1 (Onset/Mid)<br>T peak = 150.3 (endo)	T peak = 10.6 (endo)<br>T g = 15.7/16.5 (Onset/Mid)
DAPA-Cur	7.1	T g = 14.7/15.7 (Onset/Mid)<br>T g = 51.3/54.2 (Onset/Mid)<br>T peak = 160.0 (endo)	T g = 13.1/13.8 (Onset/Mid)<br>T g = 62.8/65.3 (Onset/Mid)

DAPA-Cur-LPs	14.6	T g = -6.5/-1.0 (Onset/Mid)<br>T peak = 125.8 (endo)	T g = 33.0/32.6 (Onset/Mid)
PVP-DAPA-Cur-LPs	4.3	T g = 12.7/15.6 (Onset/Mid)<br>T peak = 89.8 (endo)	T g = 13.8/14.8 (Onset/Mid)
DAPA-OA	3.7	Multiple peak<br>T peak = 38.5 (endo)<br>T peak = 53.1 (endo)<br>T peak = 126.7 (endo)<br>T m = 294.0 (endo)	T g = 62.6/64.9 (Onset/Mid)<br>T peak = 290.6 (endo)<br>T c = 274.6 (exo)
DAPA-OA-LPs	4.3	T peak = 48.2 (endo)<br>T peak = 84.0 (endo)	T g = 15.6/16.2 (Onset/Mid)
PVP-DAPA-OA-LPs	5.2	T g = 12.3/14.5 (Onset/Mid)<br>T peak = 86.9 (endo)<br>T m = 220.3 (endo)	T peak = -16.3 (endo)<br>T g = 12.6/13.9 (Onset/Mid)

Table S2. Thermogravimetric data comprising the main degradation steps, as well as weight loss (%) and the amount of the analyzed samples (mg).

**Sample**	**T onset** (°C)	**T endset** (°C)	**T peak (DTA)** (°C)	**T peak (DTG)** (°C)	**Total weight loss at 700°C (Residue), %**	**DTA characteristic (endo/exo process)**	**Mass probe (mg)**
DAPA	394.9	417.0	401.7	407.4	19.8	-endo	23.0
EMPA	323.4	444.4	156.8<br>352.2<br>418.9	352.5<br>420.8	35.1	-endo<br>-endo<br>-exo	15.5
Cur	321.0	412.2	181.0	384.0	41.1	-endo	8.6
OA	392.8	426.6	122.6<br>315.0 (mp)<br>422.0	418.6	5.6	-endo<br>-exo<br>-endo	8.8
EMPA-Cur	330.8	402.6	152.8	374.1	41.5	-endo	11.8
EMPA-Cur-LPs	multi step	447.1	130.6<br>222.0<br>424.5<br>263.0	121.9<br>261.8<br>356.2<br>423.7	21.8	-endo<br>-endo<br>-endo<br>-endo	21.2
PVP-EMPA-Cur-LPs	multi step	448.8	225.9 (small)<br>421.3<br>563.8	261.8<br>355.3<br>421.7	21.7	-endo<br>-endo<br>-endo	15.2

EMPA-OA	352.6	414.7	157.4<br>245.8<br>357.2 (mp)	367.6<br>389.3	22.8	-endo<br>- endo<br>-exo	13.1
EMPA-OA-LPs	multi step	445.0	220.7<br>271.0<br>432.7	124.3<br>267.1<br>363.1<br>417.3	22.4	-endo<br>-endo<br>-endo<br>-endo	16.5
PVP-EMPA-OA-LPs	multi step	449.8	223.6<br>263.0 (small)<br>360.3<br>436.8<br>530.8	259.4<br>364.2<br>424.8	15.6	-endo<br>-endo<br>-endo<br>-endo<br>-endo	21.2
DAPA-Cur	367.0	420.9	167.3	402.2	29.1	-endo	17.8
DAPA-Cur-LPs	multi step	446.2	132.0<br>213.3<br>268.6<br>431.9	124.7<br>262.8<br>421.6	21.0	-endo<br>-endo<br>-endo<br>-endo	17.3
PVP-DAPA-Cur-LPs	multi step	451.4	227.8<br>421.3	262.6<br>419.2	22.3	-endo<br>-endo	12.8
DAPA-OA	378.2	413.6	117.4<br>294.1<br>410.8	392.5<br>402.7	12.0	-endo<br>-endo<br>-endo	9.2
DAPA-OA-LPs	multi step	449.6	221.7<br>265.3<br>432.9	262.7<br>425.4	19.9	-endo<br>-endo<br>-endo	33.9
PVP-DAPA-OA-LPs	multi step	450.0	223.8<br>436.9	261.8<br>350.9<br>422.6	19.2	-endo<br>-endo<br>-endo	12.9
